# Dissection of miRNA-miRNA Interaction in Esophageal Squamous Cell Carcinoma

**DOI:** 10.1371/journal.pone.0073191

**Published:** 2013-09-05

**Authors:** Bingli Wu, Chunquan Li, Pixian Zhang, Qianlan Yao, Jianyi Wu, Junwei Han, Liandi Liao, Yanjun Xu, Ruijun Lin, Dawei Xiao, Liyan Xu, Enmin Li, Xia Li

**Affiliations:** 1 College of Bioinformatics Science and Technology, Harbin Medical University, Harbin, China; 2 Department of Biochemistry and Molecular Biology, The Key Laboratory of Molecular Biology for High Cancer Incidence Coastal Chaoshan Area, Shantou University Medical College, Shantou, China; 3 Institute of Oncologic Pathology, Shantou University Medical College, Shantou, China; 4 Department of Cardiothoracic Surgery, The First Affiliated Hospital, Shantou University Medical College, Shantou, China; The University of Hong Kong, China

## Abstract

The relationships between miRNAs and their regulatory influences in esophageal carcinoma remain largely unknown. Accumulated evidence suggests that delineation of subpathways within an entire pathway can underlie complex diseases. To analyze the regulation of differentially expressed miRNAs in subpathways of esophageal squamous cell carcinoma (ESCC), we constructed bipartite miRNA and subpathway networks to determine miRNA regulatory influences on subpathways. The miRNA-subpathway network indicated that miRNAs regulate numerous subpathways. Two principal biological networks were derived from the miRNA-subpathway network by the hypergeometric test. This miRNA-miRNA network revealed the co-regulation of subpathways between the upregulated and downregulated miRNAs. Subpathway-subpathway networks characterized scale free, small world, and modular architecture. K-clique analysis revealed co-regulation of subpathways between certain downregulated and upregulated miRNAs. When ESCC patients were grouped according to their expression levels of paired upregulation of miR-31 and downregulation of miR-338-3p, survival time analysis revealed a significant difference based on miR-31-miR-338-3p interaction. These findings can facilitate the understanding of the biological meaning of miRNA-miRNA interactions with either the same or opposite expression trend.

## Introduction

miRNA is a kind of single-stranded non-coding small RNA that regulates gene expression at the posttranscriptional level by base-pairing with protein-coding mRNAs [1]. The biological importance of miRNAs mainly relies on the functions of their target genes. More than ten years of investigation has confirmed that miRNAs participate in almost all aspects of important biological functions in a broad spectrum of human diseases [2]. To systematically understand miRNA function at the genome-wide level, much research has been carried out by analyzing miRNA through GO or pathway enrichment. Xu *et al.* constructed a functional miRNA–miRNA cooperation network via co-regulating functional modules. Predicted miRNA-miRNA interaction is validated by high co-expression of functional modules and negative regulation of functional modules, and the topological features of disease miRNAs in the cooperative network are distinct from non-disease miRNAs [3]. Another comprehensive analysis on miRNAs was carried out by determination of each known miRNA on biochemical pathways in the KEGG and TRANSPATH database and the Gene Ontology categories were found enriched with respect to its target genes. A strong relation to disease-related regulatory pathways has been found by investigating target pathways of miRNAs [4].

The impact of miRNA on pathways is through the regulation of its pathway member. Recent studies have shown that the damaged pathway does not necessarily occur at the overall level, but more likely strong disturbance at local area of pathway (subpathway). In recent years, more attention has been paid to subpathway, which can provide more detailed information of complex diseases in high-throughput data analysis, because critical genes may not be significantly enriched in the whole pathway, but nevertheless play key roles [5], [6]. Based on the concept of the subpathway, Li *et al.* systematically analyzed the miRNA regulatory influences to subpathways and found that a small fraction of miRNAs were global regulators, and that miRNAs co-regulate groups of subpathways with similar function. By integrating the disease states of miRNAs, Li *et al.* also found that disease miRNAs regulated more subpathways than non-disease miRNAs [7].

Esophageal cancer is the eighth most common cancer and the sixth most common cause of cancer deaths worldwide, with esophageal squamous cell carcinoma (ESCC) being the dominant type in East Asia [8]. Dozens of dysregulated miRNAs have been found in ESCC [9]. The enriched subpathways of predicted target genes of ESCC differentially expressed miRNA were considered respond to the related dysregulated disease pathways. However, there are still no reports focused on the subpathways regulation mediated by miRNA and the interactions between miRNAs in ESCC applying a network-based method. In this study, we constructed a bipartite graph of miRNA–subpathway interactions and two sub-networks (miRNA-miRNA and subpathway-subpathway) to characterize the miRNA regulatory influence on subpathways in ESCC.

## Materials and Methods

### ESCC Differentially-expressed miRNAs

The differentially expressed miRNAs used in this study were collected from the union of three earlier ESCC miRNA microarray results of our research group and other two research groups. We previously analyzed the miRNA expression profile in three pairs of ESCC tissues and matched adjacent non-cancerous tissues using a miRCURY™ locked nucleic acid (LNA) array (version 11.0 Exiqon, Denmark) and identified 33 upregulated miRNAs and 40 downregulated miRNAs, including miR-143, miR-145, miR-338-3p, miR-1261, miR-31 and miR-142-3p [10]. Subsequently, the downregulation of miR-143 and miR-145 were confirmed by QRT-PCR in 86 matched pairs of ESCC specimens [10]. In our another research, the downregulation of miR-338-3p and miR-1261 and the upregulation of miR-31 and miR142-3p were confirmed also by QRT-PCR in other 89 matched pairs of ESCC specimens [11]. Feber *et al.* analyzed 10 matched pairs of esophageal carcinoma specimens with Ambion bioarrays and reported 14 dysregulated miRNAs in ESCC, including the upregulation of miR-21 and miR-342, the downregulation of miR-203, miR-205 and let-7c [12]. Eight differentially-expressed miRNAs were also obtained from the analysis of 31 matched pairs of ESCC specimens and corresponding adjacent normal esophageal tissues by Guo *et al.* using their own designed miRNA microarray. They found upregulation of miR-25, miR-424, miR-151, miR-103 and miR-107, and downregulation of miR-100, miR-99a and miR-29c [13]. Of these miRNAs, hsa-miR-103/107 expression trends were determined by QRT-PCR in another 11 matched pairs of ESCC cases [13]. No differentially expressed miRNAs from three ESCC miRNA profiles were inconsistent, but rather were complementary. Then the 56 union differentially expressed miRNAs obtained from these three miRNA profiles were applied in this study as a dataset, many of which were verified later by other researches in ESCC by QRT-PCR (Table 1). To determine if the miRNA was related to human disease, the disease information of miRNAs was extracted from the miR2Disease database (March 2011), which contained disease-miRNA relationships extracted from literature [22].

**Table 1:** Summary of differentially-expressed ESCC miRNAs applied in this study.

miRNAs	Expressionlevel	DiseasemiRNA	Intersection of predicted target gene and ESCC DEGs	Subpathway number	ESCC profile Reference	Confirmed by QRT-PCR
hsa-miR-103	up	Yes	515	141	[13]	N/A
hsa-miR-107	up	Yes	502	152	[13]	N/A
hsa-miR-1246	up	N/A	2042	222	[10]	[14]
hsa-miR-1248	up	N/A	4057	397	[10]	N/A
hsa-miR-1280	up	N/A	762	172	[10]	N/A
hsa-miR-142-3p	up	Yes	215	134	[10] [11]	N/A
hsa-miR-142-5p	up	Yes	427	90	[10]	N/A
hsa-miR-146b-5p	up	Yes	466	143	[10]	N/A
hsa-miR-152	up	Yes	465	143	[10]	N/A
hsa-miR-15a	up	Yes	853	315	[10]	[15]
hsa-miR-424	up	Yes	846	267	[12]	N/A
hsa-miR-181a	up	Yes	835	225	[10]	N/A
hsa-miR-18b	up	Yes	235	44	[10]	N/A
hsa-miR-199a-3p	up	Yes	134	59	[10]	N/A
hsa-miR-21	up	Yes	299	65	[10] [13]	[14]
hsa-miR-22	up	Yes	308	133	[10]	N/A
hsa-miR-25	up	Yes	442	142	[10] [12]	[16]
hsa-miR-31	up	Yes	729	117	[10] [11]	[15]
hsa-miR-338-5p	up	Yes	689	40	[10]	N/A
hsa-miR-381	up	Yes	685	75	[10]	N/A
hsa-miR-491-3p	up	Yes	190	48	[10]	N/A
hsa-miR-645	up	N/A	556	89	[10]	N/A
hsa-miR-720	up	Yes	1120	194	[10]	[17]
hsa-miR-93	up	Yes	802	142	[12]	N/A
hsa-let-7c	down	Yes	619	177	[12] [13]	N/A
hsa-miR-125b	down	Yes	462	79	[10]	N/A
hsa-miR-126	down	Yes	14	87	[10]	[15]
hsa-miR-1261	down	N/A	1651	116	[10] [11]	N/A
hsa-miR-133a	down	Yes	335	63	[10]	[14]
hsa-miR-133b	down	Yes	372	66	[10]	[14]
hsa-miR-134	down	Yes	161	69	[10]	N/A
hsa-miR-143	down	Yes	299	98	[10]	[14]
hsa-miR-145	down	Yes	463	179	[10]	[14]
hsa-miR-192	down	Yes	107	60	[12]	N/A
hsa-miR-194	down	Yes	359	64	[12]	N/A
hsa-miR-200b	down	Yes	795	179	[10]	N/A
hsa-miR-200c	down	Yes	759	207	[12]	N/A
hsa-miR-203	down	Yes	1865	331	[10] [13]	[18]
hsa-miR-205	down	Yes	375	109	[12] [13]	[19]
hsa-miR-27b	down	Yes	841	219	[12]	N/A
hsa-miR-29c	down	Yes	662	229	[12]	[14]
hsa-miR-30a	down	Yes	921	216	[10]	N/A
hsa-miR-320a	down	Yes	128	82	[10]	N/A
hsa-miR-320b	down	Yes	2022	407	[10]	N/A
hsa-miR-338-3p	down	Yes	342	58	[11]	[20]
hsa-miR-378	down	Yes	175	60	[10]	N/A
hsa-miR-451	down	Yes	28	35	[10]	N/A
hsa-miR-571	down	N/A	641	134	[10]	N/A
hsa-miR-604	down	N/A	404	82	[10]	N/A
hsa-miR-617	down	N/A	767	178	[10]	N/A
hsa-miR-644	down	N/A	719	115	[10]	N/A
hsa-miR-662	down	Yes	312	131	[10]	N/A
hsa-miR-671-5p	down	N/A	1098	159	[10]	N/A
hsa-miR-891a	down	N/A	10	1	[10]	N/A
hsa-miR-99a	down	Yes	32	72	[10] [12]	[21]
hsa-miR-100	down	Yes	30	68	[10] [12]	[21]

### miRNA Target Collection and Curation

The predicted target genes of ESCC differentially expressed miRNAs were retrieved from miRecords, which stores predicted miRNA targets produced by 11 established miRNA target prediction programs, including DIANA-microT, MicroInspector, miRanda, MirTarget2, miTarget, NBmiRTar, PicTar, PITA, RNA22, RNAhybrid, and TargetScan [23]. These programs are based on different prediction algorithms, such as target site evolutionary conservation and thermodynamic stability of the RNA–RNA duplex (Text S1). Except for sites in the traditional 3′UTR, some programs could predict miRNA binding sites in the target mRNA CDS and 5′UTR. Such as RNA22 successfully predicted target sites for miR-296, miR-470 and miR-134 in the CDS regions of the Nanog, Sox2 and Oct4 mRNAs [24].

Only targets predicted by at least 4 of 11 programs and filtered by the default value of programs in miRecords were applied in the present study (Text S1, File S1). TargetScan, for example, applies context scores to consider features such as the AU content in the vicinity of the site and the position of the site within the message, to predict the function and quantitative efficacy of each site [25]. This criterion reduces the false positive rate to ensure the authenticity of miRNA target genes and subsequent analysis results to a certain extent.

Integrating both sequence information and expression profiles of miRNAs and mRNAs can potentially identify the relevant miRNA-mRNA pairs, thus facilitating interventional experiments to validate *bona fide* targets of miRNAs. Because differentially expressed miRNA might correspond with dysregulation of mRNA, mRNA expression data in ESCC were considered to ensure the tissue specificity of miRNA targets in ESCC. Six ESCC mRNA expression profile datasets (GSE17351, GSE20347, GSE29001, GSE33426, GSE33810 and GSE23400) were downloaded from NCBI GEO. Then we used fold-change analysis to identify differentially expressed genes (DEGs) from the corresponding ESCC cases and controls. Since miRNA can regulate targeted mRNA at different strengths, such as inhibiting translation, degradation and cleavage, we defined the threshold of ESCC DEGs at 1.5-fold. Next, the intersections of predicted targets from miRecords and ESCC DEGs from ESCC mRNA profile were computed for subsequent subpathway analysis for each differentially expressed miRNA, respectively. Taken together, our miRNA targets predictions strategy considers targets from multiple prediction softwares as well as ESCC mRNA expression data.

### Subpathway Analysis of miRNA Targets

In this study, the k-clique method in SubpathwayMiner R packages was applied to identify the significantly miRNA regulated subpathways based on the targets of each ESCC differentially expressed miRNA [5]. All pathways from KEGG database are considered for analysis in this study [26]. SubpathwayMiner is able to identify all subpathways based on closeness of genes in pathways through a given distance parameter k, which means that the distance among all genes within the subpathways is no greater than k. Briefly, each pathway is converted to an undirected graph with genes as nodes. After inputting the given each miRNA target genes and distance parameter k, the method can mine each subpathway and then identify statistically significantly enriched subpathways. Use of different settings of the distance parameter k allowed the identification of subpathways to become more flexible. In this study we used the default value of parameter k (k = 4). The k-clique method has been proved to be effective in subpathway identification [5]. It can identify some important subpathways which contained very important nodes (genes), such as membrane receptors or their ligands and end-points are transcriptional factors.

### miRNA-subpathway Network Generation

A pair of miRNA–subpathway regulation relationship was defined when target genes of an individual miRNA were significantly enriched in one subpathway (*P*<0.05). After assembling all significant pairs, a network of miRNA–subpathway was generated and visualized by Cytoscape, where nodes represented miRNAs or subpathways, and edges represented their regulation relationship [27]. Then an upregulated miRNA-subpathway network and downregulated network were constructed based on the expression levels of miRNA in ESCC.

### miRNA–miRNA Network and Subpathway–subpathway Network Construction

Because miRNA can regulate multiple targets and one target is probably controlled by multiple miRNAs, this pattern was applicable for miRNA in the regulation of subpathways [7]. To comprehensively understand the internal relationships of miRNAs or subpathways based on the relationship of a miRNA-subpathway in ESCC, the significances of multiple subpathways shared by two different miRNAs, and multiple miRNAs regulating two common subpathways were statistically analyzed, respectively. miRNA-miRNA networks and subpathway-subpathway networks derived from the miRNA-subpathway network were constructed using a cumulative hypergeometric distribution. The formula was as follows:
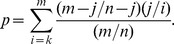



For each miRNA-miRNA pair, *n* denotes the total number of subpathways derived from the KEGG pathways, *m* represents the number of subpathways that are regulated by one differentially expressed miRNA, *i* denotes the number of subpathways that are regulated by the other differentially expressed miRNA, and *j* represents the number of overlapping subpathways that are regulated by the two miRNAs. To analyze the significance of each subpathway-subpathway pair, *n* denotes the total number of miRNAs that regulate subpathways, *m* represents the number of miRNAs that regulate one subpathway, *i* denotes the number of miRNAs that regulate the other subpathway, and *j* represents the number of overlapping miRNAs that regulate the two subpathways.

All analyses were run by the costumed R program. If the *P*-value is <0.01, the miRNA-miRNA pair or subpathway-subpathway pair was considered significant, and then the networks were also constructed and visualized by Cytoscape, in which nodes represented miRNAs or subpathways and edges represented their interactions.

### Analysis of Network Properties

The topological characteristics of networks obtained in this study were analyzed using NetworkAnalyzer, one of the Cytoscape plugins for network topological parameter analysis [28]. The edges in all networks were treated as undirected. The degree of a miRNA (or subpathway) was the number of subpathways (or miRNAs), which connected the miRNA (or subpathway) in the network. Node degree distribution *P*(*k*) is defined as the number of nodes with a degree *k* for *k* = 0, 1, 2, …. By fitting a line on datasets, such as node degree distribution data, the pattern of their dependencies can be visualized. NetworkAnalyzer considers only data points with positive coordinate values for fitting the line where the power law curve of the form *y* = *βx^a^*. The R^2^ value is a statistical measure of the linearity of the curve fit and used to quantify the fit to the power line. When the fit is good, the R^2^ value is very close to 1.

### Analysis of Cliques

CFinder software was applied to find functional communities or modules within miRNA-miRNA networks and subpathway-subpathway networks based on the Clique Percolation Method (CPM) [29]. A clique in the network was a complete subgraph in which every two miRNAs (or subpathways) were connected by an edge. Each clique must be the biggest fully connected subgraph and could be reached from each other through adjacent *k*-cliques that shared k-1 nodes [30].

### The Cluster of Two Interacting miRNAs

Expression data of interesting miRNA-miRNA interactions, detected by QRT-PCR in 89 cases of ESCC clinical samples in our previous report, were reanalyzed in this study [11]. Cluster 3.0 and Java Treeview software were applied to cluster and visualize according to the miRNA expression levels [31], [32]. Cluster 3.0 can perform a variety of types of cluster analysis on expression datasets, including hierarchical clustering, self-organizing maps, k-means clustering and principal component analysis. The k-means clustering was applied in this study.

### Survival Analysis

In our previous research, 89 matched pairs of ESCC species were collected between September of 2004 and May of 2007 in the Department of Cardiothoracic Surgery of the First Affiliated Hospital of Shantou University (Shantou, China) [11]. The follow-up of their survival time was updated to March of 2012. Then the 89 ESCC specimens were classed into two groups according to the expression of two interacting miRNAs clustering clusters result from above. The survival of grouped ESCC patients was analyzed by Kaplan–Meier analysis and the log-rank test using SPSS 13.0 (SPSS Inc, Chicago, IL).

## Results

### Subpathways of Differentially-expressed miRNAs in ESCC

**Figure 1:**
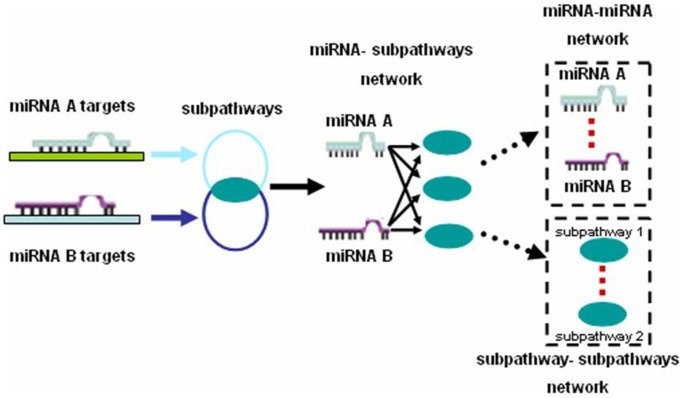
Schematic presentation of the analysis workflow to construct the miRNA-subpathway, miRNA-miRNA and subpathway-subpathway sub-networks. miRNA target genes were subjected to subpathway enrichment analysis to generate a miRNA-subpathway network. Then two sub-networks were constructed by the hypergeometric test.

The workflow to construct networks is shown in Figure 1. We initially collected 56 differentially expressed miRNAs from three ESCC miRNA microarray expression profiles, including 24 upregulated miRNAs and 32 downregulated miRNAs (Table 1). To identify whether these miRNAs were related to human disease, disease information of miRNAs was extracted from the miR2Disease database (March 2011), a manually curated database that provides a comprehensive record of miRNA deregulation involved in various human diseases, including cancers [22]. According to the miR2disease report, 44 miRNAs out of the total 56 miRNAs in this study were reported in at least one kind of human cancer. This indicates most of the differentially expressed miRNAs were closely related to human cancers and also might be critical to ESCC. The target genes of these miRNAs were retrieved from miRecords predicted by at least 4 of 11 programs and filtered by the default value of programs in miRecords (File S1). To minimize the false positives resulting from the computational prediction of miRNA targets, and to build a high-confidence resource for miRNA target analysis, ESCC mRNA DEGs were obtained by analysis of six GEO ESCC mRNA microarray profiles. The intersections of predicted targets and ESCC DEGs were retained for subsequent subpathway analysis. The miRNA expression trends, the numbers of presumed target genes and subpathway are shown in Table 1.

In total, we obtained 35942 miRNA-target regulations for these 56 miRNAs. miRNA-subpathway networks were constructed based on target gene enrichment analysis by the SubpathwayMiner package. In the miRNA-subpathway network, a miRNA and a subpathway were connected if the target genes of this miRNA were significantly enriched in this subpathway (*P*<0.05). To ensure the reliable of the targets, genes predicted by at least 4 of 11 programs in miRecords were remained, and the tissue specific of mRNA expression in ESCC has also been considered. Then these reliable targets of each miRNA were used to identify significant subpathways. This subpathway mining algorithm has been successfully to find biological significance disease-related pathways. For example, local region of tyrosine metabolic pathway was found closely related with the development of lung cancer [5]. So the subpathways we identified by subpathwayMiner though targets of miRNA are strong disordered local regions related to ESCC. As a result, 7677 significant pairs of miRNA-subpathways were found, containing 1245 unique subpathways. Interestingly, the upregulated miRNA-subpathway network comprised 27% of the subpathways derived from the KEGG human disease pathways database, whereas the downregulated miRNA-subpathway network identified comprised 26% of the subpathways derived from the human disease pathways database. These results indicate that the dysregulation miRNAs might also play significant and extensive roles in the initiation or development of ESCC through the regulation numerous disease pathways.

### miRNA-subpathway Network and its Properties

**Figure 2:**
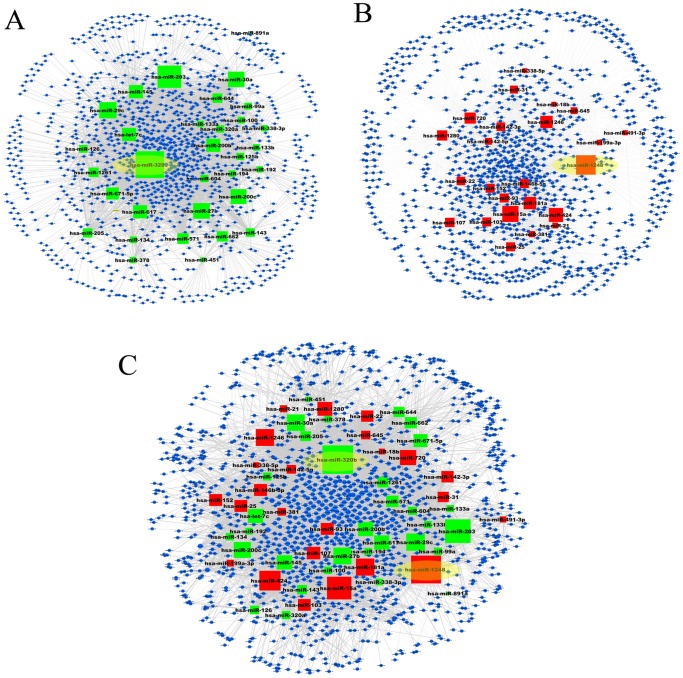
Graphic representation of three miRNA-subpathway networks. (**A**) Downregulated miRNA-subpathway network. (**B**) Upregulated miRNA-subpathway network. (**C**) Total miRNA-subpathway network. Nodes colored in green are downregulated miRNA, and red nodes are upregulated miRNAs. Blue nodes represent the subpathways. The size of the miRNA nodes correspond to the node degree (the number of subpathways that miRNA connected). *P*-value strength is represented by edge line width, with wider edges representing more significant interactions. Hsa-miR-320b and hsa-miR-1248 had the biggest degree are shaded in yellow.

The miRNA-subpathway network was constructed and visualized by Cytoscape (Figure 2). The total miRNA-subpathway network contained 7677 significant interactions between 56 miRNA nodes and 1244 unique subpathway nodes (Figure 2A, File S2). The upregulated miRNA-subpathway network contained 24 miRNA nodes and 976 subpathway nodes, generating 3548 miRNA-subpathway interactions (Figure 2B), and there were 32 miRNA nodes and 1041 unique subpathway nodes, linked by 4129 edges in the downregulated miRNA-subpathway network (Figure 2C). In the upregulated miRNA-subpathway network, hsa-miR-1248 has the largest number of subpathways, while hsa-miR-320b regulates the largest number of subpathways in the downregulated miRNA-subpathway network (Table 1, Figure 2). Compared with the results of retrieving target genes only form TargetScan (data not shown), hsa-miR-15a and hsa-miR-424 are still the second and third miRNA have greatest degree in upregulated miRNA-subpathway network, while hsa-miR-27b remains the fourth miRNA has greatest degree in downregulated miRNA-subpathway network. In comparing subpathways of hsa-miR-1248 and hsa-miR-320b, there were 8, 5 and 19 overlapping subpathways in the MAPK signaling pathway, Wnt signaling pathway, and Pathways in cancer, respectively (File S2). This shows that many overlapping subpathways are regulated by miRNAs with opposite expression.

**Figure 3:**
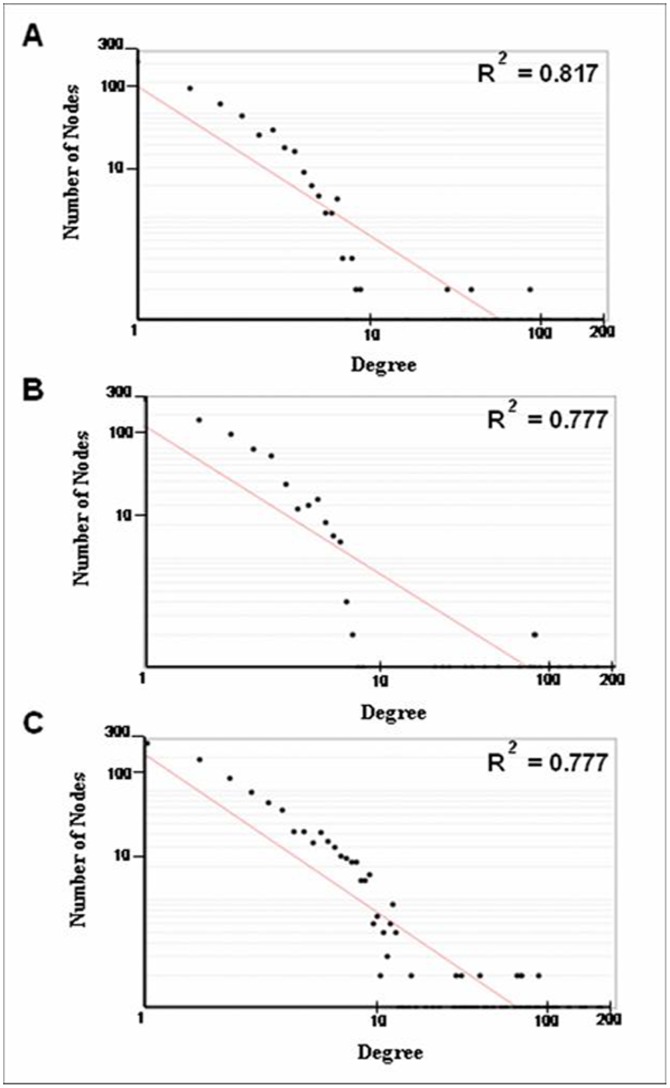
Power law of node degree distribution for the miRNA-subpathway networks. (**A**) Degree distribution of the downregulated miRNA-subpathway network. (**B**) Degree distribution of the upregulated miRNA-subpathway network. (**C**) Degree distribution of the total miRNA-subpathway network.

We then analyzed the network properties of these three miRNA-subpathway networks. The distributions of node degree approximately followed power law distributions, with an R^2^ = 0.817, 0.777 and 0.777, respectively (Table 2, Figure 3A). Thus, these three miRNA-subpathway networks were scale-free, which is one of most important characteristics of true complex biological networks [33]. These results suggest that only a few subpathway nodes are linked by many miRNAs, and that a few miRNA nodes act as hubs with a large number of links to subpathway nodes (Table S2). Compared with the results of only obtaining target genes form TargetScan (data not shown), the parameters of network properties do not change greatly (Table 2). These indicate that our analysis method was reliable.

**Table 2:** Network parameters of miRNA-subpathway and subpathway-subpathway networks.

	Nodes	Edges	*y* = *βx^a^*	R2	Correlation
**miRNA-subpathway network**					
Total miRNA	1300	7677	y = 207.49*x* ^−1.084^	0.777	0.971
Upregulated miRNA	1000	3548	y = 158.94*x* ^−1.039^	0.777	0.967
Downregulated miRNA	1073	4129	y = 184.42*x* ^−1.082^	0.817	0.989
**Subpathway-subpathway network**					
Total miRNA	817	4143	y = 462.59*x* ^−1.428^	0.852	0.866
Upregulated miRNA	362	869	y = 133.43*x* ^−1.261^	0.652	0.947
Downregulated miRNA	451	1057	y = 293.56*x* ^−1.574^	0.767	0.865

### MiRNA-miRNA and Subpathway-subpathway Network Generation

**Figure 4:**
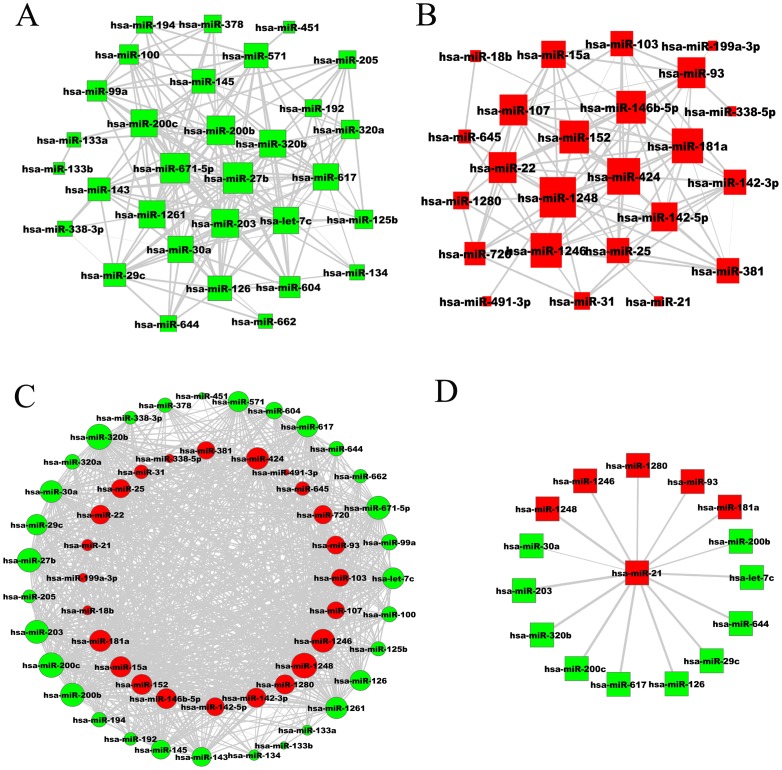
Visualization of miRNA-miRNA networks by Cytoscape. (**A**) Downregulated miRNA-miRNA network. (**B**) Upregulated miRNA-miRNA network. (**C**) Total miRNA-miRNA network. (**D**) The miRNA-miRNA interaction of hsa-miR-21. Green nodes represent downregulated miRNAs, while upregulated miRNAs are colored red. The size of the miRNA nodes corresponds to the node degree (the number of miRNAs that are connected). *P*-value strength is represented by edge line width, with wider edges representing more significant interactions.

To understand the internal relationships of dysregulated miRNAs and subpathway relationships in an ESCC background, two biologically relevant sub-networks, the miRNA-miRNA network and subpathway-subpathway network, derived from the miRNA-subpathway network, were constructed. In the miRNA-miRNA network, two miRNAs as two nodes were defined as connected if they significantly co-regulated common subpathways, as analyzed by the hypergeometric test in which subpathways from both miRNAs were significantly enriched (*P*≤0.01) (File S3). The total miRNA-miRNA network contained 790 pairs miRNA-miRNA interactions of 56 unique miRNAs (Figure 4C).

Previous research has emphasized the co-regulation of functional modules and subpathways by the cooperation of multiple miRNAs [3], [7]. In addition, another kind of miRNA-miRNA interaction appeared between miRNAs with opposite expression trends regulating the same molecules, such as mRNAs and pathways, generating reverse biological effects. For example, it has been reported that adipogenic differentiation is impaired by miR-369-5p, whereas such differentiation is highly increased by miR-371. DNMT3A and DNMT3B are up-regulated by miR-371, whereas DNMT3A is down-regulated by miR-369-5p. This result proves miR-369-5p and miR-371 are up-stream regulators of adipogenic differentiation with opposite expression levels [34]. In this study, many overlapping subpathways were regulated by miRNAs with opposite expression, such as for hsa-miR-1248 and hsa-miR-320b, as described above. To separately detect how ESCC-upregulated and -downregulated miRNAs regulate the subpathways, and identify interactions between the ESCC-upregulated and -downregulated miRNAs, ESCC-upregulated and -downregulated miRNAs were constructed in two sub-networks. The upregulated miRNA-miRNA network contained 24 miRNAs and 103 edges, while there were 31 miRNAs and 190 edges in the downregulated miRNA-miRNA network (Figure 4A and 4B). We found that the edges of the total miRNA-miRNA network were greatly increased compared to that of either the upregulated or downregulated miRNA-miRNA network, indicating increasing numbers of interactions between some upregulated and downregulated miRNAs (Table S2). For example, upregulated miR-21 had only 1 edge in the upregulated miRNA-miRNA network, but 15 edges in the total miRNA-miRNA network, in which the ratio of between upregulated and downregulated miRNAs was 5∶10 (Figure 4D). This indicates certain miRNAs are inclined to interact with miRNAs in an opposing manner.

Similarly, in the subpathway-subpathway network, a subpathway pair was defined if they were significantly co-regulated by two common miRNAs as also calculated by the hypergeometric test (*P*≤0.001) (File S4). There were 817 unique subpathways and 4134 edges in the total subpathway-subpathway network (Figure S1C). On the other hand, the downregulated subpathway-subpathway network derived from the downregulated miRNA-subpathway network contained 457 subpathways and 1057 edges, while the upregulated subpathway-subpathway network derived from the upregulated miRNA-subpathway network contained 362 subpathways and 869 edges (Figure S1A and S1B).

We next analyzed the topological properties of three subpathway-subpathway networks by NetworkAnalyzer plugin. The degree of distribution approximately followed power law distributions with an R^2^ = 0.767, 0.652 and 0.852, respectively. This indicates the three subpathway-subpathway networks are also scale-free complex biological networks (Figure S2).

### The Clique Analysis of Networks

**Figure 5:**
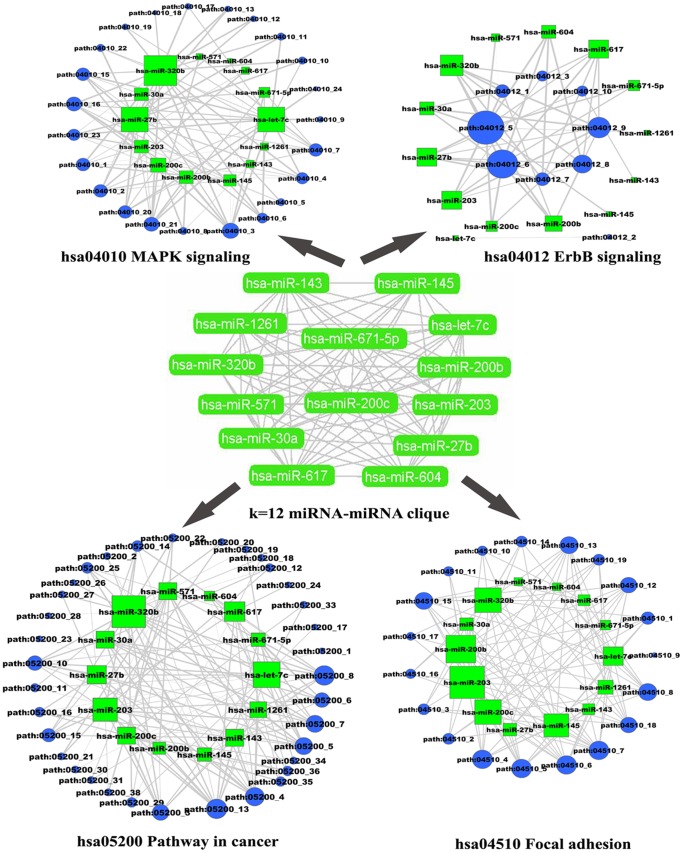
The k = 12 clique from the downregulated miRNA-miRNA network and its co-regulated subpathways. Green nodes represent downregulated miRNAs, while upregulated miRNA is colored red. The size of the miRNA nodes corresponds to the node degree. *P*-value strength is represented by edge line width, with darker edges representing more significant interactions.

For the miRNA-miRNA network, the *k*-clique method was applied to predict functional modules, which would provide important insight to the miRNAs involved. These miRNA-miRNA and subpathway-subpathway networks were mapped and colored according to the communities found by CFinder. This algorithm identifies the maximally complete subgraphs (*k-*cliques, in which any two nodes have edges) in the networks or the communities in which two *k*-cliques share exactly *k*-1 nodes [29]. In the downregulated miRNA-miRNA network, a k = 12 clique community consisted of 14 downregulated miRNAs, which included let-7c, miR-1261, miR-143, miR-145, miR-200b, miR-200c, miR-203, miR-27b, miR-30a, miR-320b, miR-571, miR-604, hsa-miR-617 and miR-671-5p (Figure 5). Ten of these 14 miRNAs were human cancer-related miRNAs. To understand how these miRNAs were connected, we surveyed the subpathways regulated by these 14 miRNAs and found that there were at least four significant pathways derivative subpathways that were co-regulated by this clique, including Pathways in cancer, Focal adhesion, and ErbB signaling pathway and MAPK signaling pathways. These four pathways were co-regulated by all 14 miRNAs of this clique (Figure 5). It has been acknowledged that these four pathways play critical roles in the development of ESCC [35], [36], [37]. Of these 14 miRNAs, 8 miRNAs (let-7c, miR-27b, miR-30a, miR-143, miR-145, miR-200b/200c and miR-203) have been found to play significant roles in ESCC, serving as prognostic markers, tumor suppressors or oncogenes [38]. Therefore, it was presumed that the all 14 miRNAs in this k = 12 clique co-operatively regulate critical pathways (subpathways) ESCC. Similarly, a k = 9 clique, also consisting of 14 miRNAs, was found by CFinder in the upregulated miRNA-miRNA network, which included miR-103, miR-107, miR-1248, miR-146b-5p, miR-152, miR-15a, miR-181a, miR-25, miR-424, miR-93, miR-1246, miR-22, miR-381 and miR-142-5p. Of these miRNAs, only miR-1246 and miR-1248 have not been reported in miR2disease. This clique co-regulates the neurotrophin and Insulin signaling pathways, which are co-regulated by 14 and 12 miRNAs from this clique (Figure S3).

**Figure 6:**
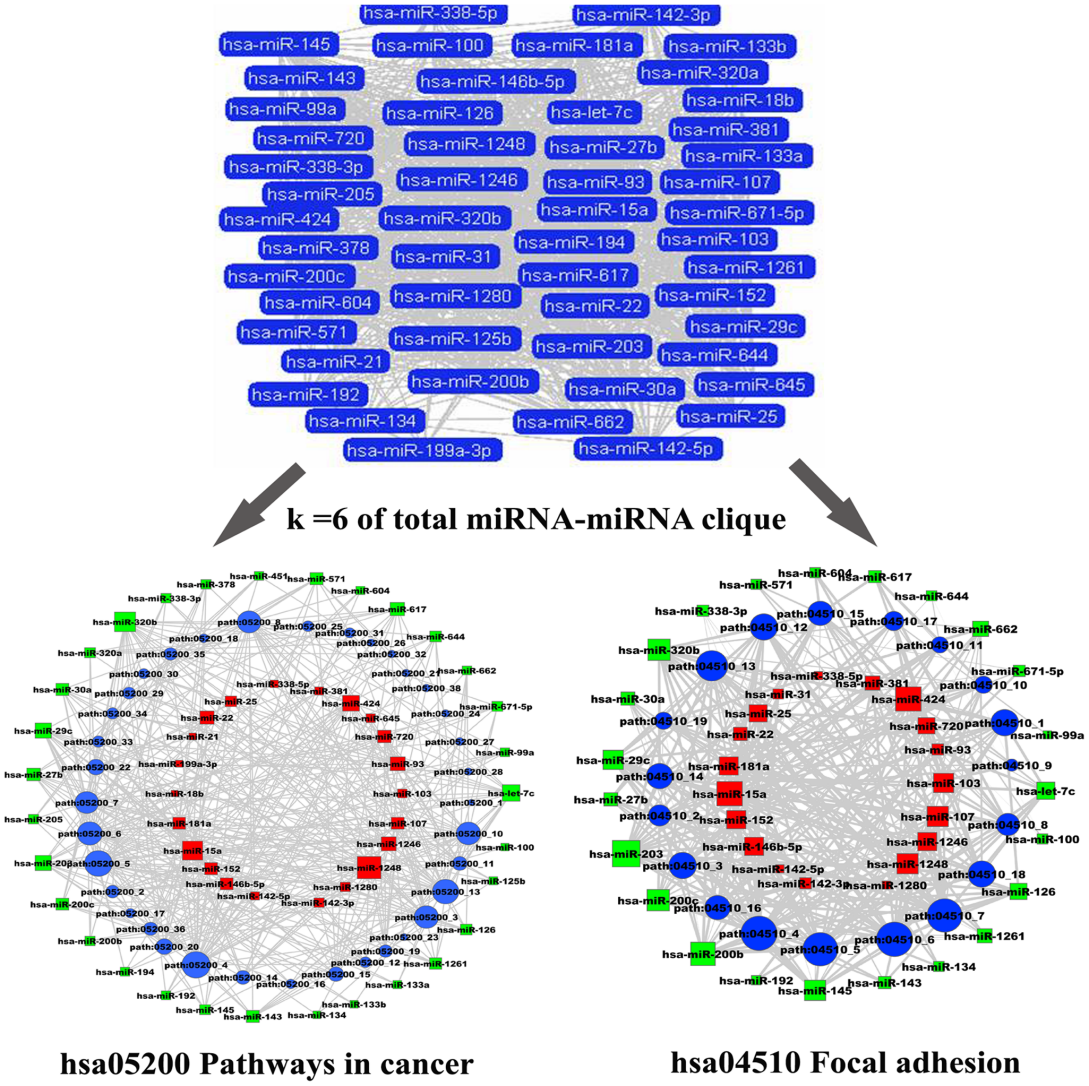
The k = 6 clique from total miRNA-miRNA and its co-regulated subpathways. Red nodes represent upregulated miRNAs, while blue nodes are downregulated miRNAs. The size of the miRNA nodes corresponds to the node degree. *P*-value strength is represented by edge line width, with wider edges representing more significant interactions.

To also test if there was an antagonistic effect between the dysregulated miRNAs, k-clique analysis was also performed on the total miRNA-miRNA network. An interesting k = 6 clique was found, which consisted of 53 miRNAs (Figure 6). To our surprise, two significant pathways were also co-regulated by 12 miRNAs of this clique. For the Pathways in cancer, it was co-regulated by 31 downregulated miRNAs, and 21 upregulated miRNAs. The Focal adhesion pathway was also co-regulated by 22 downregulated miRNAs and 19 upregulated miRNAs (Figure 6). These results indicate the subsequent biological effects depend on the extent regulated by the dysregulated miRNAs (downregulated miRNA and upregulated miRNA).

### Effect of miRNA-miRNA Interaction on ESCC Patient Survival

**Figure 7:**
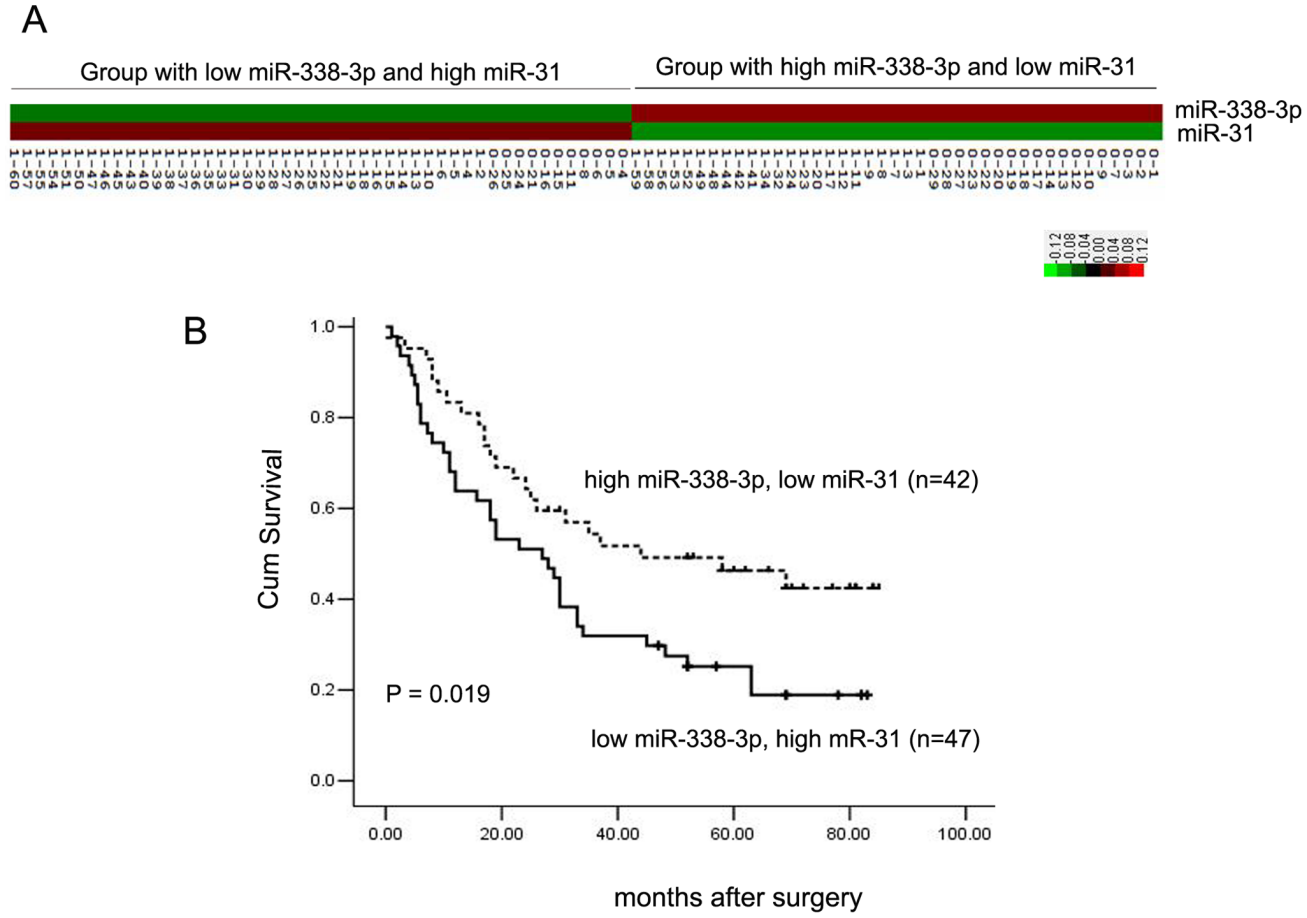
ESCC patient clusters and survival analysis. (**A**) The cluster of miR-31 and miR-338-3p in 89 ESCC patients. The prefix 0 represents deceased ESCC patients, while the prefix 1 represents living ESCC patients. (**B**) Survival of grouped ESCC patients is analyzed by Kaplan-Meier analysis and the log-rank test.

Since close connections were found in the miRNA-miRNA network, especially in the total miRNA-miRNA network, certain miRNAs interacted with other miRNAs in opposing fashion. This phenomenon was also found in the *k*-clique analysis of the miRNA-miRNA network. These results revealed the existence of miRNA-miRNA interactions between the miRNAs with opposite expression changes. To test if this type of miRNA-miRNA interaction affects the survival of ESCC patients, the expression of the miR-31 and miR-338-3p interacting pair (*P* = 0.0057, Supplementary file S3) detected by QRT-PCR in 89 matched pairs of ESCC clinical samples reported previously were clustered using Cluster 3.0 [11], [29]. One critical reason to choose this miRNA pair is that they have opposite expression trends in ESCC. The expression level of miR-31 was increased 1.58-fold while miR-338-3p was decreased 0.88-fold [11]. The cluster result showed that the 89 ESCC patients could be classified into two groups according to the expression levels of miR-31 and miR-338-3p (Figure 7A). One group contained 42 patients with high miR-338-3p and low miR-31 expression levels, while the other group was comprised of 47 patients with low miR-338-3p and high miR-31 expression levels. Survival analysis of the patients by Kaplan–Meier analysis and the log-rank test showed that the group with a higher expression level of miR-31 and lower expression level of miR-338-3p had shorter survival, compared to the group with a lower expression level of miR-31 and higher expression level of miR-338-3p (Figure 7B). These results suggest that certain miRNA-miRNA interactions between miRNAs with reverse expression trends might play a significant role in the initiation and development of ESCC, and ESCC patient survival.

## Discussion

ESCC is one of the most deadly malignant tumors in the world, with an overall 5-year survival rate less than 20% [39]. miRNA has been proven as the most important post-transcriptional regulator in the initiation and development of a variety of tumors, including ESCC. However, a system-wide level analysis of miRNA in ESCC is not currently available. Accumulated evidence proves that miRNAs regulate diverse biological pathways [40]. Previous studies demonstrate that a subpathway-based approach is more precise and flexible in annotation and identification of pathways. Thus, in this study the rules of miRNA regulatory influences on subpathways are explored in ESCC.

First, differentially expressed miRNAs were mined from three ESCC miRNA expression profiles, and their presumed target genes, obtained from the intersections of predicted targets and ESCC DEGs, were subjected to subpathway analysis to generate a miRNA-subpathway network. Hsa-miR-320b and hsa-miR-1248 had the highest degree, meaning that it regulates the largest number of subpathways in the miRNA-subpathway network, respectively. However, detailed biological functions of these two miRNA have yet to be reported. Hsa-miR-203 has the third highest degree. miR-203 represses TP63 expression at the posttranscriptional level and inhibits the proliferation of ESCC cells through the TP63-mediated signal pathway [41]. In the miRNA-subpathway network, some subpathways are uniquely regulated by certain miRNAs (Table S1). For example, KEGG pathway path:00020 (Citrate cycle (TCA cycle)) can only be regulated by hsa-let-7c, which is involved in essential amino acid synthesis. Zhang *et al.* report significant changes of TCA cycle in esophageal cancer patients compared with the healthy controls using both ^1^H NMR and UHPLC to detect the metabolomics of esophageal cancer blood serum [42]. This indicates that certain differentially expressed miRNAs might play specific roles in ESCC through the regulation of unique subpathways.

We constructed two biologically relevant networks derived from the miRNA-subpathway, the miRNA-miRNA network and subpathway-subpathway networks to provide complementary information to miRNA regulatory influences on subpathways, and allow us to understand the interactions between miRNAs or between subpathways. Our results indicate that the three kinds of miRNA-subpathway networks are true complex biological networks, rather than random networks [43].

Xu *et al.* demonstrated the miRNA-miRNA cooperation network based on Gene Ontology and protein–protein interaction networks [3], and Li *et al.* analyzed miRNA-miRNA interaction based on classification of disease and non-disease subpathways [7]. Moreover, it has been shown that the combination of several miRNAs may work together to affect multiple target genes in the same or different biological pathways [44]. Similar results are found in this study. For example, a k = 12 clique community, consisting of 14 downregulated miRNAs, is found in the downregulated miRNA-miRNA network, and at least four significant pathways derivative subpathways are co-regulated by this clique, including Pathways in cancer, Focal adhesion, and ErbB signaling, and the MAPK signaling pathway. Also, a k = 9 clique is found in the upregulated miRNA-miRNA network. miRNAs in this clique co-regulate Neurotrophin signaling pathway and Insulin signaling pathways. These results suggest that cooperative miRNA-miRNA interactions with the same changes in expression level are also critical in ESCC.

However, the effect of miRNA-miRNA interaction between miRNAs opposite changes in expression level has not been emphasized in previous network research. In this study, multiple evidence supports miRNA-miRNA interaction between miRNAs with opposite trends in expression in ESCC. First, the edges of the total miRNA-miRNA network are greatly increased compared to that of either upregulated or downregulated miRNA-miRNA networks. This might partly result from the addition of miRNA nodes, but also from the miRNA-miRNA interactions with opposite expression levels. We find upregulated hsa-miR-21 interacts with more downregulated miRNAs than upregulated miRNAs in the total miRNA-miRNA network. It should also be pointed out that hsa-miR-21 is the most upregulated miRNA in our ESCC miRNA expression profile, increasing 24.5-fold on average [10]. This indicates that hsa-miR-21 might interact with certain downregulated miRNAs, whose downregulation might facilitate upregulated hsa-miR-21 to promote the initiation or development of ESCC. Recent research shows that hsa-miR-21 plays critical roles in ESCC. Upregulation of hsa-miR-21 promotes the proliferation, migration and inhibition of apoptosis of ESCC cells through activating the ERK1/2/MAPK pathway, while its knockdown suppresses cell growth, invasion and induced apoptosis by targeting FASL, TIMP3 and RECK genes [45], [46]. To our great interest, we find hsa-miR-21 interacts with hsa-miR-203 (*P* = 0), which is the most downregulated miRNA, with an average decrease of 4.4-fold according to our previous ESCC miRNA expression profile [10]. Moreover, TP63, a target of hsa-miR-203, has also been reported to be regulated by miR-21 in glioblastoma [47]. These results indicate that most downregulated and most upregulated miRNAs extensively interact in the same biological event by targeting the same genes, or even subpathways.

To further understand the biological effect of miRNA-miRNA, with opposite changes in expression levels, on the clinicopathology of ESCC, 89 matched pairs of ESCC clinical samples reported previously were clustered and classified into two groups according to the expression level of the miR-31-miR-338-3p miRNA-miRNA pair. Survival analysis shows the ESCC patient group with a higher expression level of miR-31 and lower expression level of miR-338-3p had a shorter survival time than the group with a lower expression level of miR-31 and higher expression level of miR-338-3p. These results suggest that the interaction between miRNAs with reverse expression levels have a significant impact on the survival of ESCC patients. In contrast, in our previous results, miR-31 and miR-338-3p did not show any significance when using the expression levels of these two miRNAs to classify and analyze the survival of ESCC patients [11]. Nevertheless, the significant biological effect of both miRNAs on ESCC patient survival time was revealed when the same ESCC patients were grouped and analyzed according to the clustering of miR-31-miR-338-3p interaction. It has been previously reported that miR-31 is up-regulated in ESCC tissues and serum. ESCC patients with high-levels of serum miR-31 also have a poorer prognosis in relapse-free survival [48]. Recently, miR-338-3p has also been proven to be downregulated in ESCC, and its aberrant expression increases the risk of ESCC [20]. Our research reveals significant biological effects of miR-31-miR-338-3p interaction that simultaneously occur in ESCC patients.

In summary, based on subpathway analysis of miRNA target genes, we define the interactions between dysregulated miRNAs with the same expression trend or opposite expression trend in ESCC. Our results indicate that the biological effects of miRNA-miRNA interaction might not only reflect common targets and common pathways, but also the clinical pathology of ESCC.

## Supporting Information

Figure S1 **The visualization of subpathway-subpathway networks in Cytoscape.** (**A**) Downregulated subpathway-subpathway network. (**B**) Upregulated subpathway-subpathway network. (**C**) Subpathway-subpathway network of total miRNA. The size of the subpathway nodes corresponds to the node degree (the number of subpathways connected). *P*-value strength is represented by edge line width, with wider edges representing more significant interactions.
(TIF)

Figure S2 **Power law of node degree distribution for the subpathway-subpathway networks.** (**A**) Degree distribution of the downregulated subpathway-subpathway network. (**B**) Degree distribution of the upregulated subpathway-subpathway network. (**C**) Degree distribution of the total subpathway-subpathway.
(TIF)

Figure S3 **The k = 9 clique from the upregulated miRNA-miRNA network and its co-regulated subpathways.** The size of the miRNA nodes corresponds to the node degree. *P*-value strength is represented by edge line width, with wider edges representing more significant interactions.
(TIF)

Table S1 **Unique subpathways regulated by dysregulated miRNAs.**
(DOC)

Table S2 **Degrees of miRNAs in the total miRNA-subpathway and miRNA-miRNA networks.**
(DOC)

File S1 **The miRNAs target genes retrieved from miRecords predicted by at least 4 of 11 programs.**
(XLS)

File S2 **The subpathways of miRNA target genes.**
(XLS)

File S3 **miRNA-miRNA network.**
(XLS)

File S4 **Subpathway-subpathway network.**
(XLS)

Text S1 **The 11 algorithms in miRecords apply different weighted and ranked systems for the predicted target genes.**
(DOC)
