# Supplementary figures and images for: Dissection of miRNA-miRNA Interaction in Esophageal Squamous Cell Carcinoma
Source: PLoS One. 2013 Sep 5;8(9):e73191. doi: 10.1371/journal.pone.0073191 (PMC3764179; doi:10.1371/journal.pone.0073191)

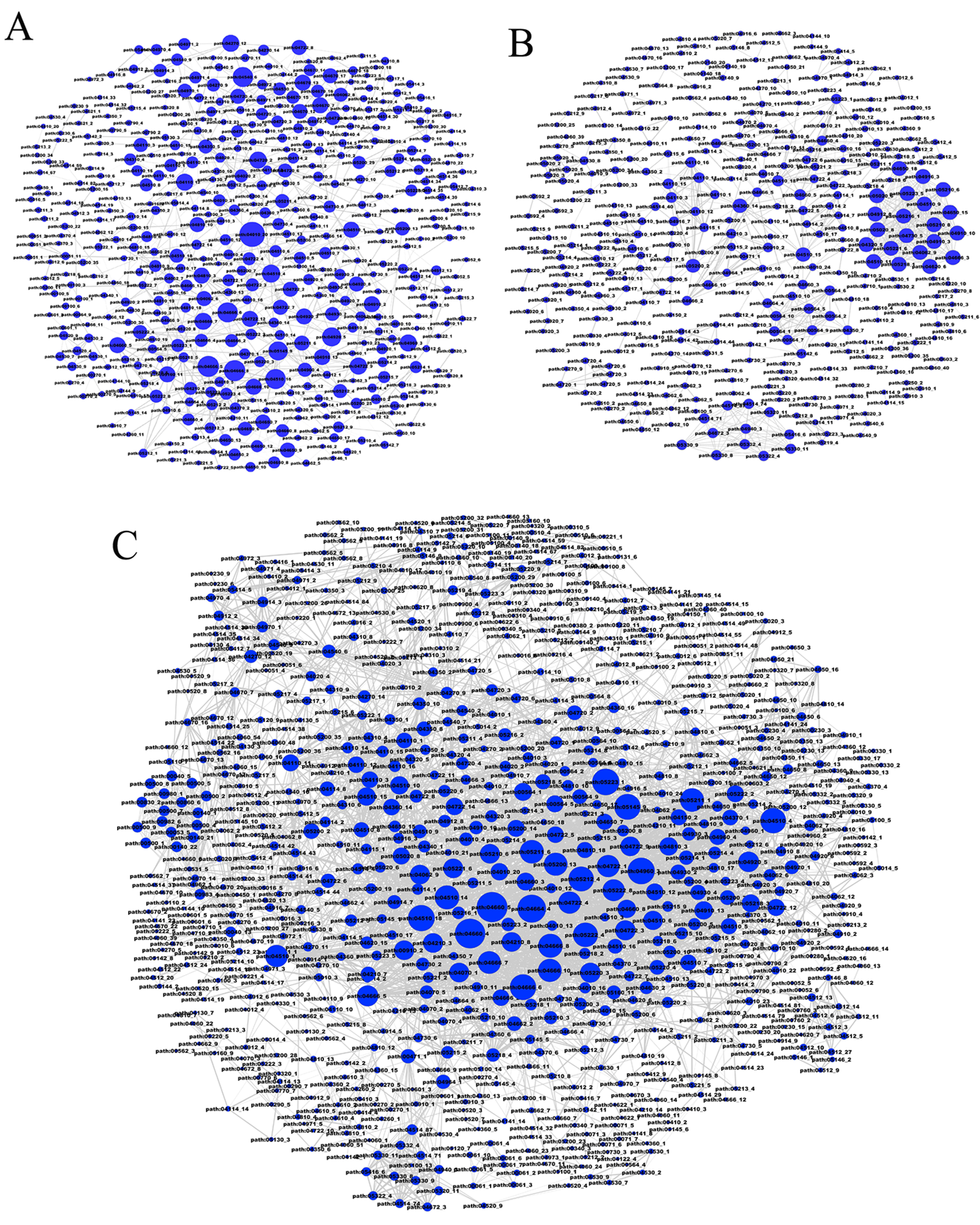

Supplement: Figure S1 — The visualization of subpathway-subpathway networks in Cytoscape. (A) Downregulated subpathway-subpathway network. (B) Upregulated subpathway-subpathway network. (C) Subpathway-subpathway network of total miRNA. The size of the subpathway nodes corresponds to the node degree (the number of subpathways connected). P-value strength is represented by edge line width, with wider edges representing more significant interactions. (TIF) [file pone.0073191.s001.tif]

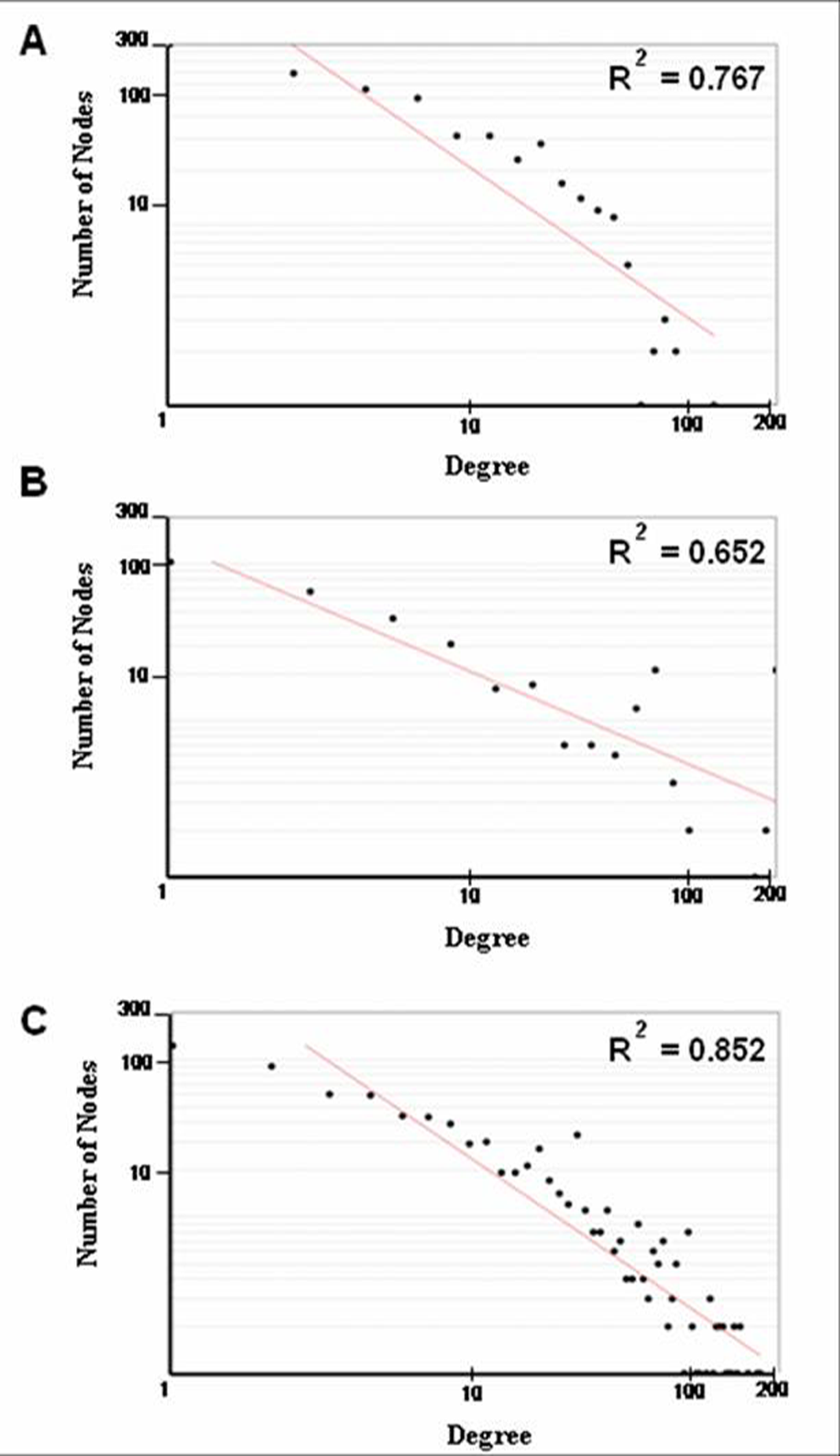

Supplement: Figure S2 — Power law of node degree distribution for the subpathway-subpathway networks. (A) Degree distribution of the downregulated subpathway-subpathway network. (B) Degree distribution of the upregulated subpathway-subpathway network. (C) Degree distribution of the total subpathway-subpathway. (TIF) [file pone.0073191.s002.tif]

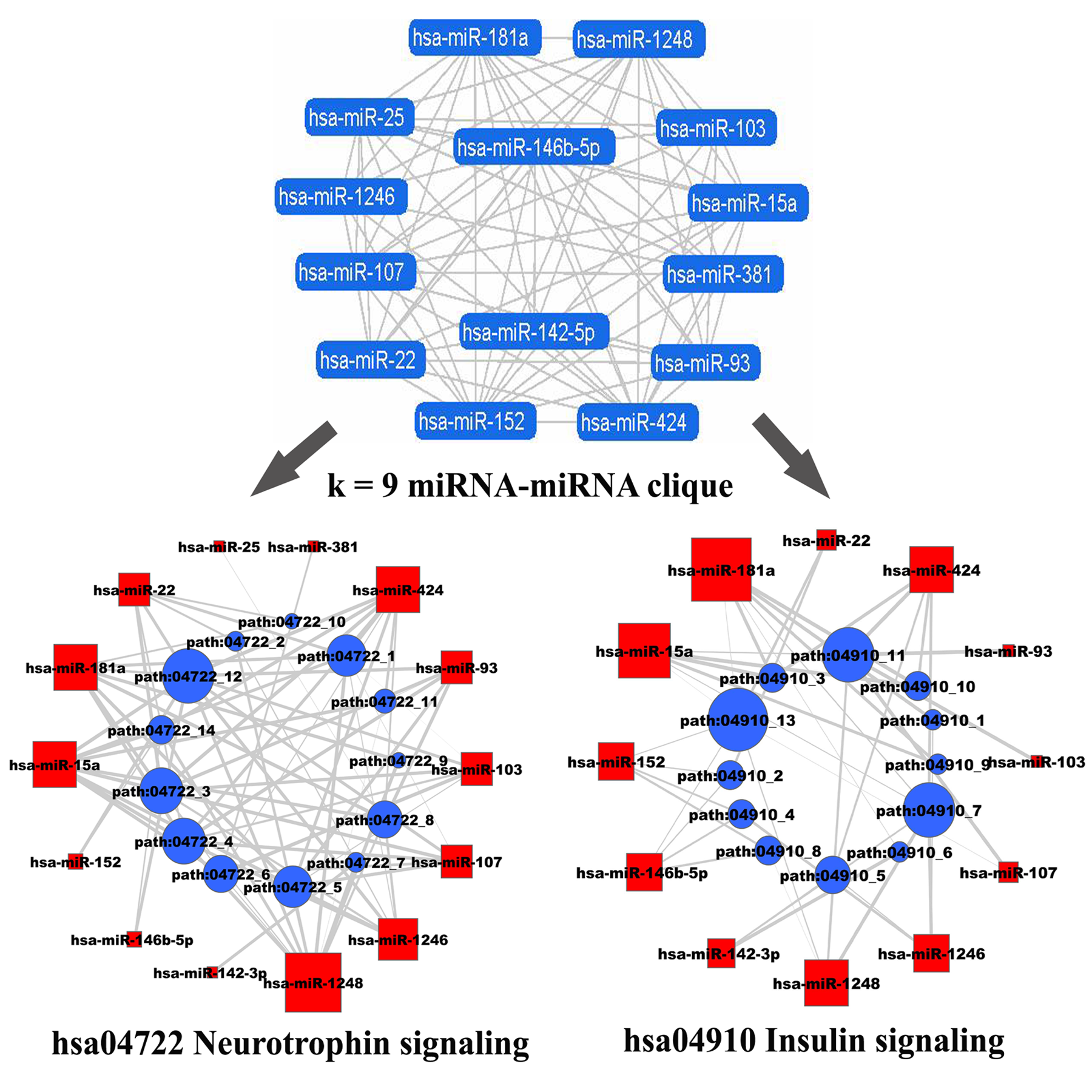

Supplement: Figure S3 — The k = 9 clique from the upregulated miRNA-miRNA network and its co-regulated subpathways. The size of the miRNA nodes corresponds to the node degree. P-value strength is represented by edge line width, with wider edges representing more significant interactions. (TIF) [file pone.0073191.s003.tif]
