# Supplementary material for: Dissection of miRNA-miRNA Interaction in Esophageal Squamous Cell Carcinoma
Source: PLoS One. 2013 Sep 5;8(9):e73191. doi: 10.1371/journal.pone.0073191 (PMC3764179; doi:10.1371/journal.pone.0073191)
Supplement: Table S1 — Unique subpathways regulated by dysregulated miRNAs. (DOC) [file pone.0073191.s004.doc]

| **Table S1.** Unique subpathways regulated by dysregulated miRNAs. | | |
| --- | --- | --- |
| **miRNA** | **subpathways** | **KEGG pathways name** |
| Downregulated miRNA | | |
| hsa-let-7c | path:00020_1 | Citrate cycle (TCA cycle) |
|  | path:00020_2 |  |
|  | path:00020_7 |  |
|  | path:05012_3 | Parkinson's disease |
| hsa-miR-145 | path:00130_8 | Ubiquinone and other terpenoid-quinone biosynthesis |
|  | path:04742_2 | Taste transduction |
| hsa-miR-194 | path:04710_1 | Circadian rhythm - mammal |
|  | path:04710_2 |  |
| hsa-miR-203 | path:00563_2 | Glycosylphosphatidylinositol (GPI)-anchor biosynthesis |
|  | path:00970_1 | Aminoacyl-tRNA biosynthesis |
| hsa-miR-29c | path:00561_1 | Glycerolipid metabolism |
|  | path:00561_2 |  |
| hsa-miR-30a | path:00430_2 | Taurine and hypotaurine metabolism |
| hsa-miR-320b | path:00120_10 | Primary bile acid biosynthesis |
|  | path:00260_7 | Glycine, serine and threonine metabolism |
|  | path:00910_2 | Nitrogen metabolism |
| hsa-miR-571 | path:00232_1 | Caffeine metabolism |
|  | path:00232_2 |  |
| hsa-miR-617 | path:00830_1 | Retinol metabolism |
|  | path:00830_3 |  |
| hsa-miR-644 | path:00670_1 | One carbon pool by folate |
| hsa-miR-662 | path:05330_11 | Allograft rejection |
|  | path:05330_12 |  |
|  | path:05330_3 |  |
|  | path:05330_8 |  |
|  | path:05330_9 |  |
|  | path:05332_4 | Graft-versus-host disease |
| Upregulated miRNAs | | |
| hsa-miR-107 | path:04973_1 | Carbohydrate digestion and absorption |
| hsa-miR-1246 | path:00072_1 | Synthesis and degradation of ketone bodies |
|  | path:00350_10 | Tyrosine metabolism |
|  | path:00350_11 |  |
|  | path:00350_13 |  |
|  | path:00650_1 | Butanoate metabolism |
|  | path:00650_6 |  |
|  | path:04142_1 | Lysosome |
| hsa-miR-1248 | path:00100_2 | Steroid biosynthesis |
|  | path:00100_3 |  |
|  | path:00100_5 |  |
|  | path:00534_1 | Glycosaminoglycan biosynthesis - heparan sulfate |
| hsa-miR-1280 | path:00740_1 | Riboflavin metabolism |
|  | path:04950_2 | Maturity onset diabetes of the young |
|  | path:05150_13 | Staphylococcus aureus infection |
| hsa-miR-22 | path:04621_3 | NOD-like receptor signaling pathway |
| hsa-miR-31 | path:00380_2 | Tryptophan metabolism |
| hsa-miR-338-5p | path:00900_4 | Terpenoid backbone biosynthesis |
|  | path:00900_6 |  |
| hsa-miR-720 | path:00565_3 | Ether lipid metabolism |
|  | path:00590_1 | Arachidonic acid metabolism |
