# Supplementary material for: Dissection of miRNA-miRNA Interaction in Esophageal Squamous Cell Carcinoma
Source: PLoS One. 2013 Sep 5;8(9):e73191. doi: 10.1371/journal.pone.0073191 (PMC3764179; doi:10.1371/journal.pone.0073191)
Supplement: Table S2 — Degrees of miRNAs in the total miRNA-subpathway and miRNA-miRNA networks. (DOC) [file pone.0073191.s005.doc]

| **Table S2.** Degrees of miRNAs in the total miRNA-subpathway and miRNA-miRNA networks. | | | | | |
| --- | --- | --- | --- | --- | --- |
| **Upregulated miRNAs** | | | **Downregulated miRNAs** | | |
| miRNA | miRNA-  subpathway network | miRNA-miRNA network | miRNA | miRNA-  subpathway network | miRNA-  miRNA network |
| hsa-miR-103 | 141 | 29 | hsa-let-7c | 177 | 38 |
| hsa-miR-107 | 152 | 29 | hsa-miR-125b | 79 | 22 |
| hsa-miR-1246 | 222 | 43 | hsa-miR-126 | 87 | 36 |
| hsa-miR-1248 | 397 | 48 | hsa-miR-1261 | 116 | 339 |
| hsa-miR-1280 | 172 | 34 | hsa-miR-133a | 63 | 12 |
| hsa-miR-142-3p | 134 | 34 | hsa-miR-133b | 66 | 6 |
| hsa-miR-142-5p | 90 | 31 | hsa-miR-134 | 69 | 13 |
| hsa-miR-146b-5p | 143 | 36 | hsa-miR-143 | 98 | 33 |
| hsa-miR-152 | 143 | 36 | hsa-miR-145 | 179 | 33 |
| hsa-miR-15a | 315 | 37 | hsa-miR-192 | 60 | 18 |
| hsa-miR-424 | 267 | 40 | hsa-miR-194 | 64 | 23 |
| hsa-miR-181a | 225 | 39 | hsa-miR-200b | 178 | 44 |
| hsa-miR-18b | 44 | 10 | hsa-miR-200c | 207 | 47 |
| hsa-miR-199a-3p | 59 | 49 | hsa-miR-203 | 331 | 43 |
| hsa-miR-21 | 64 | 16 | hsa-miR-205 | 60 | 19 |
| hsa-miR-22 | 133 | 33 | hsa-miR-27b | 219 | 44 |
| hsa-miR-25 | 142 | 32 | hsa-miR-29c | 229 | 37 |
| hsa-miR-31 | 117 | 21 | hsa-miR-30a | 216 | 40 |
| hsa-miR-338-5p | 40 | 8 | hsa-miR-320a | 82 | 23 |
| hsa-miR-381 | 75 | 30 | hsa-miR-320b | 407 | 48 |
| hsa-miR-491-3p | 48 | 2 | hsa-miR-338-3p | 58 | 19 |
| hsa-miR-645 | 89 | 21 | hsa-miR-378 | 60 | 22 |
| hsa-miR-720 | 194 | 32 | hsa-miR-451 | 35 | 4 |
| hsa-miR-93 | 142 | 31 | hsa-miR-571 | 134 | 36 |
|  |  |  | hsa-miR-604 | 82 | 28 |
|  |  |  | hsa-miR-617 | 178 | 38 |
|  |  |  | hsa-miR-644 | 115 | 23 |
|  |  |  | hsa-miR-662 | 131 | 20 |
|  |  |  | hsa-miR-671-5p | 159 | 43 |
|  |  |  | hsa-miR-891a | 1 | 0 |
|  |  |  | hsa-miR-99a | 72 | 25 |
|  |  |  | hsa-miR-100 | 68 | 24 |
