# Supplementary material for: Dissection of miRNA-miRNA Interaction in Esophageal Squamous Cell Carcinoma
Source: PLoS One. 2013 Sep 5;8(9):e73191. doi: 10.1371/journal.pone.0073191 (PMC3764179; doi:10.1371/journal.pone.0073191)
Supplement: Text S1 — The 11 algorithms in miRecords apply different weighted and ranked systems for the predicted target genes. (DOC) [file pone.0073191.s010.doc]

The 11 algorithms in miRecords apply different weighted and ranked systems for the predicted target genes.

1. **TargetScan/TargetScanS**: It applies context scores to consider features such as the AU content in the vicinity of the site and the position of the site within the message, to predict the function and quantitative efficacy of each site [1,2].

### PicTar: PicTar computes the maximum likelihood score (PicTar score) that RNA sequence is targeted by combinations of microRNAs from the search set when compared to background and the individual probability Pi for each subsequence of the RNA sequence to be bound by a miRNA (only the nuclei for the binding sites are depicted) [3].

1. **miRanda**: The algorithm takes into account G-U wobble pairs, allows moderate insertions and deletions and, importantly, uses a weighting scheme that rewards complementarity at the 5' end of the miRNA, as observed in known miRNA:target-mRNA duplexes. In addition, it have applied position-specific empirically defined rules, and the result is a score (S) for each detected complementarity match between a miRNA and a potential target gene [4].
2. **DIANA-microT**: The total predicted score of a miRNA:target gene interaction is the weighted sum of conserved and unconserved MREs of a gene. It also provide a signal-to-noise ratio (SNR) and a precision score specific for each interaction that can be used as a helpful confidence estimation of the ‘correctness’ and the false positive rate of each predicted target [5].
3. **MicroInspector**: The program generates a list of possible target sites, sorted by free energy values. Adaptation of temperature and free energy settings, followed by visual inspection of secondary structures allows a detailed analysis [6].
4. **RNAhybrid**：Using the Dynamic Programming technique, the program calculates the MFE hybridizations of all possible start positions in the miRNA and in the target. It complements the optimization of miRNA/target duplexes by a thorough statistical analysis of minimum free energies (MFEs). RNAhybrid normalizes MFEs with the sequence lengths of miRNAs and targets, and model such normalized MFEs as extreme value distributed. The parameters of these distributions are estimated specifically for every miRNA with RNAcalibrate and are subsequently used to assign *p*-values to normalized MFEs [7].
5. **RNA22**：It is a pattern-based approach for the discovery of microRNA binding sites and their corresponding microRNA/mRNA complexes. RNA22 has high sensitivity, is resilient to noise, and can be applied to the analysis of any genome without requiring genome-specific retraining [8].
6. **miTarget**: miTarget is a support vector machine (SVM) classifier for miRNA target gene prediction. It uses a radial basis function kernel as a similarity measure for SVM features, categorized by structural, thermodynamic, and position-based features [9].
7. **MirTarget2**: A scoring system was developed to assign scores to all 3′UTRs with seed matching sites. A small fraction of 3′UTRs had multiple candidate sites, and all sites in one UTR were combined to compute the UTR score as the following [10].
8. **NBmiRTar**: The NBmiRTar classifier assigns a score to each miRNA: mRNA candidate and classifies it into one of the two predefined classes: the positive class (target) and the negative class (non-target). It allows the user to obtain his prediction by inputting the miRNA(s) and 3′UTR sequences with option of applying any of the three filters (miRanda score filter, folding free energy and Naive Bayes score filter) [11].

**Supplementary references**

1. Lewis BP, Shih IH, Jones-Rhoades MW, Bartel DP, et al. (2003) Prediction of mammalian microRNA targets. Cell 115:787-98.
2. Lewis BP, Burge CB, Bartel DP (2005) Conserved seed pairing, often flanked by adenosines, indicates that thousands of human genes are microRNA targets. Cell 120:15-20.
3. Krek A, Grün D, Poy MN, Wolf R, Rosenberg L, et al. (2005) Combinatorial microRNA target predictions. Nat Genet 37:495-500.
4. John B, Enright AJ, Aravin A, Tuschl T, Sander C, et al. (2004) Human MicroRNA targets. PLoS Biol 2:e363.
5. Kiriakidou M, Nelson PT, Kouranov A, Fitziev P, Bouyioukos C, et al. (2004) A combined computational-experimental approach predicts human microRNA targets. Genes Dev 18:1165-78.
6. Rusinov V, Baev V, Minkov IN, Tabler M (2005) MicroInspector: a web tool for detection of miRNA binding sites in an RNA sequence. Nucleic Acids Res 33(Web Server issue):W696-700.
7. Rehmsmeier M, Steffen P, Hochsmann M, Giegerich R (2004) Fast and effective prediction of microRNA/target duplexes. RNA 10:1507-17.
8. Miranda KC, Huynh T, Tay Y, Ang YS, Tam WL, et al. (2006) A pattern-based method for the identification of MicroRNA binding sites and their corresponding heteroduplexes. Cell 126:1203-17.
9. Kim SK, Nam JW, Rhee JK, Lee WJ, Zhang BT (2006) miTarget: microRNA target gene prediction using a support vector machine. BMC Bioinformatics 7:411.
10. Wang X, El Naqa IM (2008) Prediction of both conserved and nonconserved microRNA targets in animals. Bioinformatics 24:325-32.
11. Yousef M, Jung S, Kossenkov AV, Showe LC, Showe MK (2007) Naïve Bayes for microRNA target predictions--machine learning for microRNA targets. Bioinformatics 23:2987-92.
